# Vasoplegic Syndrome and Anaesthesia: A Narrative Review

**DOI:** 10.4274/TJAR.2023.221093

**Published:** 2023-08-18

**Authors:** Begüm Nemika Gökdemir, Nedim Çekmen

**Affiliations:** 1 Department of Anaesthesiology and Reanimation, Başkent University Faculty of Medicine, Ankara, Turkey

**Keywords:** Anaesthesia management, diagnostic and therapeutic approach, perioperative period, vasoplegic syndrome

## Abstract

Vasoplegic syndrome (VS) is defined as low systemic vascular resistance, normal or high cardiac output, and resistant hypotension unresponsive to vasopressor agents and intravenous volume. VS is a frequently encountered complication in cardiovascular and transplantation surgery, burns, trauma, pancreatitis, and sepsis. The basis of the pathophysiology is associated with an imbalance of vasodilator and vasoconstrictive structure in vascular smooth muscle cells and is highly complex. The pathogenesis of VS has several mechanisms, including overproduction of iNO, stimulation of ATP-dependent K+ channels and NF-κB, and vasopressin receptor 1A (V1A-receptor) down-regulation. Available treatments involve volume and inotropes administration, vasopressin, methylene blue, hydroxocobalamin, Ca++, vitamin C, and thiamine, and should also restore vascular tone and improve vasoplegia. Other treatments could include angiotensin II, corticosteroids, NF-κB inhibitor, ATP-dependent K+ channel blocker, indigo carmine, and hyperbaric oxygen therapy. Despite modern advances in treatment, the mortality rate is still 30-50%. It is challenging for an anaesthesiologist to consider this syndrome’s diagnosis and manage its treatment. Our review aims to review the diagnosis, predisposing factors, pathophysiology, treatment, and anaesthesia approach of VS during anaesthesia and to suggest a treatment algorithm.

**Main Points**

• Vasoplegic syndrome (VS) is defined as low systemic vascular resistance, normal or high cardiac output, and resistant hypotension unre-sponsive to vasopressor agents and intravenous (IV) volume.

• VS is an essential complication in cardiac and transplantation surgery, burns, trauma, pancreatitis, and sepsis.

• VS is related to highly increased perioperative morbidity and mortality.

• Overproduction of iNO, activation of ATP-dependent K^+^ channels and NF-κB, and vasopressin receptor 1A (V_1_A-receptor) downregulation are responsible for the pathogenesis of VS.

• Rapid identification and diagnosis of at-risk patients should implement an optimal therapeutic approach.

• Treatment strategies involve IV administration of volume and inotropes, vasopressin, methylene blue, angiotensin II, corticosteroids, hydroxocobalamin, Ca++ vitamin C, and thiamine, and should also restore vascular tone and improve vasoplegia.

## Introduction

Vasoplegic syndrome (VS) is defined as low systemic vascular resistance (SVR), normal or high cardiac output (CO), and resistant hypotension unresponsive to vasopressor agents and intravenous (IV) volume. VS should be considered when perioperative refractory hypotension develops in cardiovascular and transplantation surgery.^1^

Normal organ and tissue functions are provided by adequate tissue perfusion and oxygen.

Requires adequate mean arterial pressure (MAP), CO, heart rate (HR), stroke volume, CO, and SVR for average systemic circulation. Physiologically, factors that determine hemodynamics are MAP, SVR, and CO. It is defined by the formula [SVR=(MAP-RAP)/CO].^1,2^ Studies recommend this component for the diagnosis of VS:

1) Low SVR (SVR<700 dyn s cm^-5^);

2) Systolic blood pressure <90 mmHg or severe hypotension (MAP) <60 mmHg refractory to vasopressors use (norepinephrine = NE);

3) Unchanged or high CO;

4) Cardiac index (CI >2.2 L min^-1^ m^-2^);

5) Refractory to adequate volume expansion (Table 1).^1,3,4^

VS can also lead to significant organ dysfunction, manifested by systemic tissue hypoperfusion and high lactate, due to insufficient cellular oxygen utilization.^5^

Although no rate is specified in the literature on the overall incidence of VS, it has been reported in all age groups and various clinical circumstances, such as sepsis, cardiopulmonary bypass (CPB), anaphylaxis (including protamine reactions), hemodialysis, all shock states, cardiac arrest, blood transfusion, transplantation, burns, trauma, and pancreatitis.^3,5,6,7,8^ VS is a common complication of major cardiovascular surgery: The incidence of VS in patients undergoing CPB is between 9% and 44%,^8^ and it accounts for 4.6% of all forms of circulatory shock.^6,7,8^ The incidence of VS after heart transplantation ranged between 8.8 and 54% and 42% after pulsatile left ventricular assist device (LVAD) implantation.^3,5,6^ The most typical cause of VS in the intensive care unit (ICU) is sepsis. The incidence depends upon the definition used and the patient population under consideration.^5,8,9,10^

Despite modern advances in treatment, the mortality rate is still 30-50%. VS can lead to systemic tissue hypoperfusion due to insufficient cellular oxygen use. As a result, it leads to multi-organ failure, especially acute kidney injury, resulting in longed duration hospital and ICU stays and raised costs.^6,7,8,10,11,12^ Thus, VS is associated with increased morbidity in the perioperative period.^7,8,12,13,14,15,16^ It is challenging for an anaesthesiologist to consider this syndrome’s diagnosis and manage its treatment.

Our review aims to review the diagnosis, predisposing factors, pathophysiology, treatment, and anaesthesia approach of VS during anaesthesia and to suggest a treatment algorithm.

### Pathogenesis in Vasoplegic Syndrome

**Figure 1:**
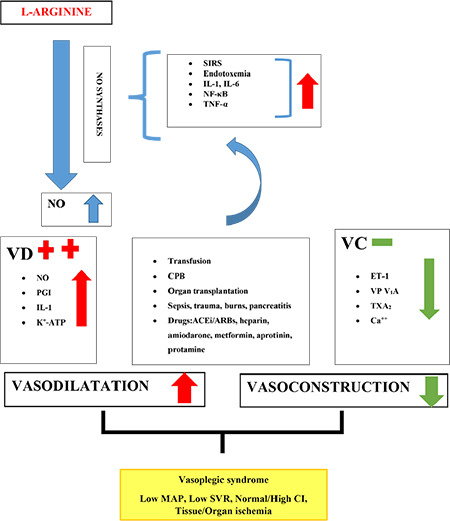
The basic pathogenesis of vasoplegic syndrome. SIRS, systemic inflammatory response syndrome; IL, interleukin; NF-κB, nuclear factor kappa B; TNF-α, tumor necrosis factor alpha; NO, nitric oxide; PGI, prostacyclin; ET, endothelin; VP, vasopressin V1A; TXA2, thromboxane A2; VD, vasodilatation; VC, vasoconstriction; MAP, mean arterial pressure; SVR, systemic vascular resistance; CI, cardiac index.

The pathogenesis of VS has several mechanisms, including overproduction of iNO, stimulation of ATP-sensitive K^+^ channels and NF-κB, and vasopressin receptor 1A (V_1_A-receptor) down-regulation (Figure 1).^5,8,9,10,11,12^ The basis of the pathophysiology is associated with an imbalance of vasodilator and vasoconstrictive structure in VSMC and is highly complex and multifactorial.^17^ The release of intracellular Ca^++^ from the sarcoplasmic reticulum and an increase in extracellular Ca^++^ via voltage-sensitive channels triggers the contraction of vascular smooth muscle cells (VSMC). The contractile activity of VSMC determines SVR. Intrinsic and extrinsic mechanisms regulate vascular tone. Intrinsic regulators include NO, prostanoids, oxygen free radicals, endothelin-1 (ET-1), and non-endothelial factor as K^+^ channel hyperpolarisation, vasoactive metabolites as acidosis, hypoxia, hydrogen peroxide physiologically active substance as (such as serotonin, prostaglandins, thromboxane A_2_ (TXA_2_), bradykinin).^5,17,18^ Extrinsic regulators include epinephrine, vasopressin, angiotensin II, and sympathetic control. NO is an important mediator that regulates vascular function produced from endothelial L-Arginine via NO synthase. NO causes relaxation and vasodilation in VSMC.

In addition, NO prevents coagulation by inhibiting platelet aggregation and adhesion.^18^ Prostacyclin (PGI_2_) is mainly synthesized in vascular endothelial cells and VSMC. PGI_2_ is synthesized from membrane phospholipids via COX-2 and PGI synthase. PGI_2_’s primary functions inhibit platelet aggregation, effect relaxation, and cause vasodilation via cAMP-protein kinase A in VSMC^19^, significantly increasing inflammation.^5,17,19^ Overproduction of PGI_2_ causes the induction of pro-inflammatory cytokines such as tumor necrosis factor-alpha (TNF-α), interleukin-1 β (IL-1 β), IL-6, pathogen-associated molecular pattern molecules, and lipopolysaccharide.^5,8,10,11^ ET-1 activates endothelin A receptors in VSMC released from the endothelium, producing a rise in intracellular Ca^++^ and acting as a potent vasoconstrictor. However, ET-1 leads to an increased pro-inflammatory process in inflammatory stress conditions by activating many signals.^20^ TXA_2_ is a short-lived prostanoid that increases vasoconstriction and platelet aggregation.^21^ As a result, the main reason for the pathogenesis of VS is the decrease in ET-1 against excessive NO release. With the deterioration of this balance, refractory hypotension develops with changes in SVR and CO, and VS occurs with heterogeneous events (Figure 1).^5,8,9,10,11,12^

### Perioperative Risk Factors in Vasoplegic Syndrome

Many risk factors increase the development of VS. The most important risk factors are blood transfusion, CPB, transplantation, burns, trauma, pancreatitis, sepsis, and drugs (Table 2).^3,5,8,11,12,13,14,15,16^

### a) Blood transfusion

The first known and essential risk factor for VS is blood transfusion. Transfusion of blood products contributes to the development of VS by inducing inflammation. In addition, blood products used in the treatment of anemia may worsen the condition by activating inflammatory pathways due to their immunomodulatory effects.^22^

### b) CPB surgery

VS is frequently seen in 9-44% of patients during or after CPB surgery.^8^ The pathophysiologic basis of VS is also based on patient characteristics, comorbidities, and surgical procedures. The contact of blood elements, which starts with surgical trauma, with the foreign surface of the pump system initiates inflammation. In addition, many systems are activated with ischemia/reperfusion injury, hypothermia, endotoxemia, surgical stress, and exacerbation of inflammation with anaesthesia.^5,8^ As a result, cytokines (IL-1β, IL-6, TNF-α), complement system, coagulation-fibrinolysis cascade, neurohumoral (bradykinin-kallikrein-kinin system) and endothelial (iNO, PGI_2_) factors, and cellular immune system are induced causing systemic inflammatory response syndrome.^8,9,10^ Adjuvant factors other than NO cause VS development by activating ATP-dependent K^+^ channels in myocytes and stimulating the release of endothelium-derived hyperpolarizing factor.^5,8,9,10^

Ultimately, VS is a transient vascular dysfunction caused by inflammation, vasodilation, refractory to fluid replacement, and vasopressors.^5^ This causes hypoperfusion and metabolic acidosis. If not treated adequately, it results in high mortality and morbidity.^5,6,7^ If VS continues for 36-48 hours, the prognosis gets worse, and the mortality rate increases by 16-27%.^6,7,8^

Risk factors for VS during CPB include male gender, higher body mass index, elderly (>65 years), anemia, high EuroSCORE, ejection fraction (EF) <35%, myocardial ischemia, diabetes mellitus, dialysis-dependent renal failure, LVAD use, prolonged of CPB, hypotension soon after the onset of CPB, angiotensin-converting enzyme inhibitors (ACEi) and angiotensin receptor blockers (ARBs) use, infected endocarditis, and the use of inodilators (milrinone, dobutamine, levosimendan) in the perioperative period (Table 3).^8,11,12,13,14,15,16^

The cardiac anaesthesiologist should evaluate essential risk factors that worsen VS, such as acid-base and fluid-electrolyte disorders, hypothermia, and hypoxia before surgery. Cardiovascular surgery requires a multidisciplinary and comprehensive assessment and management approach.

### c) Organ transplantation

Liver and kidney transplantation surgery has a significantly higher risk of developing VS in the perioperative period, which is usually confronted following graft reperfusion.^23,24^ VS occurs in 2% to 20% of patients who undergo liver transplantation (LT) and may result in mortality.^23^ The cause of VS during LT is multifactorial. The development of VS during the new hepatic phase of LT is an essential challenging clinical scenario, requiring rapid diagnosis and treatment and appropriate management.^23,24^

Hepatic failure is characterized by activation of the renin-angiotensin-aldosterone system and arteriolar vasodilation despite the excessive release of endogenous catecholamines. Hyperdynamic circulation with increased CO, increased HR and SVR, hypotension, and hypovolemia are seen mainly due to vasodilation in the splanchnic circulation in hepatic failure. Portal hypertension causes a significant endothelial change followed by endothelial cell stretching and sheer stress, leading to a rise in endogenous NO, carbon monoxide, and hydrogen sulfide (H_2_S) release. This results in VSMC relaxation, decreased SVR, vasodilatation, as well as a deficiency of vasopressin. This condition contributes to VS.^25^ Additionally, it contributes to the development of VS during LT in situations such as acidosis, hypothermia, hypocalcemia, bleeding, hyperkalemia, and pre-operative renal dysfunction.^3,5^ This hemodynamic deterioration becomes more pronounced during LT, usually requiring a vasopressor, and puts the LT patient at risk.^23^

Transesophageal echocardiography (TEE) can guide evaluation information on cardiovascular function and intravascular volume status, exclude pulmonary embolism and ischemia, evaluate ventricular function, and assess SVR.^26^ Considering the diagnosis of VS, necessary vasopressor and other treatments should be promptly started. However, the main goal is to maintain adequate organ perfusion and hemodynamics.^3,5^

Suppose volume replacement and resistance to vasoactive drugs and hemodynamic instability continue after hemodynamic, laboratory, TEG findings, and TEE evaluation. Vasoactive drug selection should be made carefully based on clinical findings. Considering the relative deficiency in liver failure, vasopressin should be considered the first choice.^23,27^ MB may be an essential option for us in transplant surgery in post-reperfusion syndrome (PRS) and VS. If PRS continues after graft reperfusion, VS should be considered.^27^

### d) Trauma, burns, pancreatitis, and sepsis

Necessary conditions such as polytrauma, burns, severe pancreatitis, and sepsis cause hypermetabolism, systemic inflammation, and significant tissue damage, thus predisposition to developing VS and organ dysfunction.^28,29^

### e) Pharmacologic agents

There are many different categories of drugs used to be associated with VS. Among these, the most common drugs causing are ACEi/ARBs.^3^ Drugs such as metformin, protamine, aprotinin, heparin, amiodarone, nitrates, Ca^++^ channel blockers, phosphodiesterase inhibitors, and β- blockers^3,8,10,15^ have been reported to cause VS potentially but have not been fully proven; however, administration of ACEi 24 hours before surgery is a reported risk factor for VS with an incidence of 26.9%. It should always be kept in mind that VS may develop in the presence of important risk factors in patients using these drugs and undergoing major surgery.^30^

### Monitoring and TEE in Vasoplegic Syndrome

Central venous catheters and intraoperative TEE are routinely used in cardiovascular and transplantation surgery. TEE provides comprehensive information about the heart’s anatomy, contraction, ventricular function, and wall motions. If VS is suspected, the first action is to exclude pathologies such as ventricular and valve dysfunction, wall motion abnormalities, ischemia, pulmonary embolism, volume status, or pericardial tamponade. The diagnosis of VS should be confirmed by evaluating these findings observed in TEE and the hemodynamic data obtained from PICCO.^3,5,26^ After hemodynamic, laboratory, TEG findings, TEE evaluation, volume replacement, and vasoactive drugs should be considered if hemodynamic instability continues.

### Management of Vasoplegic Syndrome

**Figure 2:**
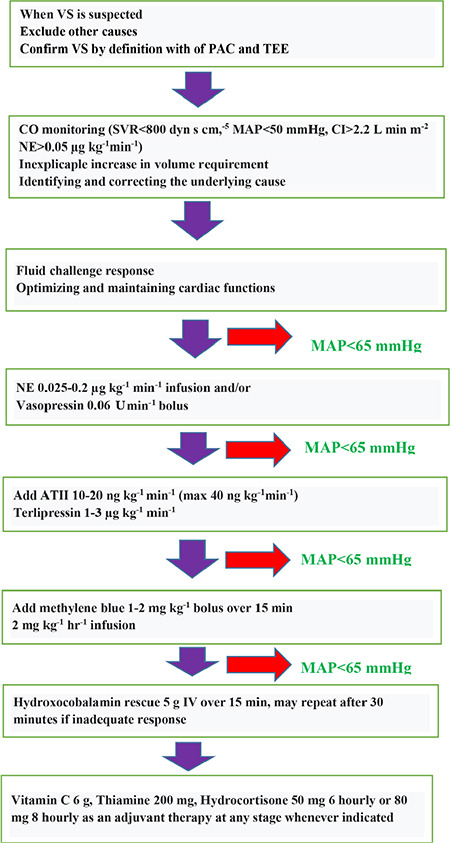
A systematic practical approach to the management of vasoplegic syndrome during anesthesia. VS, vasoplegic syndrome; PAC, pulmonary artery catheter; TEE, transesophageal echocardiography; CO, cardiac output; SVR, systemic vascular resistance; MAP, mean arterial pressure; CI, cardiac index; NE, norepinephrine.

Early recognition of VS is essential for correct treatment and management. That is, the clinician should always consider and confirm VS in the presence of low SVR, hypotension, and standard/high CO.^5,6,11,12^ Current treatment strategies include volume and vasopressors administration vasopressin, MB, high dose hydroxocobalamin, angiotensin II, vitamin C, thiamine, and corticosteroids. Other treatments could involve NO inhibitors, ATP-dependent K^+^ channel blockers, NF-κB inhibitors, indigo carmine, and hyperbaric oxygen therapy.^11,12,31^ When we suspect VS in our clinic during CPB, organ transplantation, and other surgeries, the diagnosis and treatment are summarized in Figure 2 as indicated and recommended in the literature.^10,11,12^

### Vasoactive Drugs

VS management is based on restoring vascular tone. For this reason, vasoactive agents are preferred first in the treatment. These agents act on various receptors to elevate MAP and SVR. If MAP remains low (<60 mmHg) despite adequate fluid resuscitation, vasopressors should be initiated. It is suggested to start NE or vasopressin as first-line agents to provide systemic perfusion pressure.^5,11,12^ These vasoactive agents are of three types depending on their mechanism of action. 1) Catecholamines, 2) Non-catecholamines (Hormones), 3) NO inhibitors (Table 4).^11,12^

### 1. Catecholamines

Catecholamines form the basis of treatment. These drugs induce the contraction of VSMC by stimulating adrenergic receptors and increasing cytosolic Ca^++^.^5^ This group includes NE, epinephrine, phenylephrine, and dopamine. These drugs are agents that increase MAP and should be considered first.^5,10^

### 2. Non-catecholamines (Hormones)

Vasopressin, terlipressin, and angiotensin II are vasopressors that increase non-adrenergic cytosolic calcium. They are more effective than catecholamines in providing vascular tone without affecting L-type Ca^++^ channels.^11,12^

### Volume resuscitation

Determination of volume response is essential in treating VS. In major surgeries, hypovolemia due to bleeding, hypoperfusion, and acidosis may deepen, and VS may be more mortal. According to hemodynamic data, appropriate fluid and blood products should be used cautiously to correct hypovolemia due to bleeding. Optimal perfusion and oxygenation as adequate as possible for tissues and organs should be ensured. However, excessively aggressive volume resuscitation (>20-30 mL kg^-1^) causes excessive vascular endothelial fluid shear stress, unnecessary systemic pressures, and increased extravascular lung water. Meanwhile, both hypovolemia and hypervolemia are associated with increased mortality and morbidity, and both should be avoided as much as possible.^32,33^

### 3. Nitric oxide inhibitors

### Methylene blue (MB)

MB should be considered an alternative treatment drug in cases resistant to fluid and inotrope in VS.^34^ MB reduces NO production by inhibiting iNO synthase and guanylate cyclase, potentially reducing NO concentration and other physiological stress. As a result, MB inhibits NO-mediated phosphorylation of myosin and associated vasodilation, includes coronary vasoconstriction, reduces splanchnic blood flow, and increases pulmonary vascular resistance.^32^ Additionally, MB administration has demonstrated an increase in MAP and SVR, a decrease in CI from supranormal level, lower TNF-α concentration, and a gradual reduction in vasopressors requirements.^34^ Boluses of 1-2 mg kg^-1^ at VS can usually be given over 10-20 minutes or up to 1 hour. Intravenous administration generally has a terminal half-life of 5-6 hours, and a continuous infusion of 1 mg kg^-1^ hr^-1^ after an initial bolus for up to 48-72 hours without sacrificing splanchnic perfusion may be beneficial.^11,12,32,34^ Although adverse reactions are rare with MB, they can sometimes be severe. This may exacerbate hemolytic anemia due to MB’s inhibition of pulmonary vasoconstriction, especially in patients with hypoxia and glucose-6-phosphate dehydrogenase deficiency. In addition, since MB and leucomethylene blue are excreted in the urine, they stain the urine green. MB infusion also has dose-dependent, mild to severe side effects like nausea and vomiting, chest pain, hypertension, interference with pulse oximetry readings without PaO_2_, compromised splanchnic perfusion, methemoglobinemia, hyperbilirubinemia, and serotonin syndrome.^32^ Finally, MB treatment can improve vascular tone and improve mortality and morbidity in VS.^34^

### Other Treatments

### Corticosteroids

Corticosteroids guide inflammatory tissue responses, including cytokine release and circulating immune cell function. Corticosteroids probably inhibit the arachidonic acid cascade and the NF-κB transcription factor.^35^ These activities are driven by the regulation of many intermediate pathways involving iNOS-mediated NO synthesis and COX-2 activity. Accordingly, these drugs reduce pro-inflammatory cytokine, leukotriene, and endotoxin levels, such as IL-10 increases anti-inflammatory mediators.^5,11,12,35^ Corticosteroid receptors are found in endothelial and VSMC, and under normal conditions, they increase the response to circulating catecholamines and angiotensin II.^5,35^ Presumably, corticosteroids increase the efficacy of vasopressors by increasing the sensitivity of vascular adrenergic receptors and alleviating the underlying inflammatory process that causes loss of vascular tone.^5,31,35^ In the treatment of VS, low doses of corticosteroids seem to increase the vascular response to NE.^5,11,12,31,35^ In our clinic, we give low-dose corticosteroids and inotrope in cases where VS develops.

### Vitamin C

Vitamin C (ascorbic acid) is a micronutrient that has begun to be used in the treatment of VS. Vitamin C cannot be synthesized endogenously and is an essential cofactor in the biosynthesis of endogenous catecholamines.^36^ Vitamin C deficiency in critically ill patients may worsen the clinical picture by decreasing the production of endogenous vasopressors such as NE and vasopressin.^5,36^ Vitamin C may reduce inflammation and improve microcirculation with its anti-inflammatory effects. In addition, due to antioxidative properties, vitamin C scavenges reactive oxygen species, decreases NO_S_ induction, and increases susceptibility to catecholamines by decreasing adrenergic receptors back to baseline conditions. These properties, presumably similar to corticosteroids, may restore systemic vascular tone and reduce the vasopressors needed to maintain hemodynamic goals.^7,31,36,37,38^ It has been shown that administering high-dose IV vitamin C (25 mg kg^-1^ or 1.5 g every 6 hours) can ameliorate hemodynamic changes, inflammation, and body functions in seriously ill patients, even in the absence of vitamin C deficiency.^7,37^ However, high doses of vitamin C can cause hyperoxaluria, so it should be carefully monitored.^7,36,37,38^

### Vitamin B12

Vitamin B12 (hydroxocobalamin) effectively scavenges NO released in endothelial blood vessels or diffuses into the perivascular space and reduces NO signals, resulting in reduced vasodilation. Vitamin B12 may also act as a catalyst for vitamin C, and O_2_ is applied to inactivate NO signalling in VS.^39^ Vitamin B12 has also been shown to increase the clearance of H_2_S and endothelial hyperpolarization factors. Although traditionally used in treating cyanide poisoning, the reason vitamin B12 produces raised vascular tone is unknown. A critical side effect of vitamin B12 infusion is increased MAP in patients. Although vitamin B12 can cause chromaturia, its difference from MB is that there is no risk of serotonin syndrome.^40,41^

### Non-cardiac surgery

Following major non-cardiac surgery, patients may develop hypotension due to vasodilation. Vasopressors usually need to maintain optimal MAP, with suitable resuscitation to correct hypotension. Hypotension due to sympathetic blockade often occurs in neuraxial blocks. VS should be considered when the need for volume replacement and vasopressors develops against the systemic vasodilator effects of neuraxial blockade.^42^

## Discussion

There are many studies on VS in the literature. A systematic review by Egi et al.^43^ compared multiple agents in the treatment of VS, including norepinephrine, dopamine, and phenylephrine. They suggested that no particular vasopressor was superior to the others but suggested using a second agent with a different mechanism of action in line with BP targets. Hajjar et al.^9^ in the Vasoplegic Shock After Cardiac Surgery study, compared vasopressin and norepinephrine as first-line therapy for the treatment of VS after cardiothoracic surgery, reporting a lower incidence of atrial fibrillation and acute renal failure, as a combined consequence of mortality or serious complications, in the vasopressin group.

Intraoperative MB application has been described widely in the literature. Mehaffey et al.^34^ retrospectively analyzed 118 patients who underwent CPB and were given MB in the early (intraoperative) and late (postoperative) period to prevent VS. They reported that mortality and incidence of renal failure were lower in patients who were given MB in the early period compared to those given in the late period, and the results were more positive.^34^ We do not provide MB prophylactically in CPB surgery in our clinic, but we use MB for treatment in cases where VS develops. Gunt and Çekmen^44^ reported successfully treating MB in a patient who underwent LT and developed VS. Wieruszewski et al.^38^ noted in the case series of three cardiac surgery patients that there was a decrease in vasopressor requirements in all three patients after ascorbic acid administration in the treatment of refractory VS. In two patients they reported that they did not need vasopressor support after 24 hours.

Recently, published case reports and series have shown an increase in MAP in patients with VS when hydroxocobalamin is administered at 5 g over 15 minutes.^39,45,46,47,48^ Shah et al.^39^ retrospectively examined 33 patients who had undergone cardiac surgery and reported that hydroxocobalamin administration could provide a beneficial alternative treatment for refractory hypotension and VS, but more controlled clinical studies are needed to evaluate its efficacy.

Currently, no data supports or favours non-catecholamine therapy over other therapies. A balanced approach of catecholamine and non-catecholamine treatments is crucial in managing VS. It may be by allowing more optimal doses to avoid toxicity risks. Based on the best available evidence in the literature, vasopressin may be considered the first-choice agent among non-catecholamine drugs in combination with catecholamines.^11^ Jha^49^ reported that although vasopressin is recommended for high EF patients with good heart function, NE is preferred for cardiac surgery patients with poor ventricles and low EF.

The basic approach to VS is presented in Table 4.^11,12^ Non-catecholamine agents should be started with lower catecholamine doses (0.1 µg kg^-1^ min^-1^), with vasopressin as the first-line non-catecholamine agent followed by MB. Subsequently, hydroxocobalamin and/or angiotensin II should be used when catecholamine doses are increased to 0.2 µg kg^-1^ min^-1^.^8,11,12^ Care should be taken to identify potential risk factors for intolerance or adverse reaction and to avoid or discontinue the drug with an adverse reaction. In addition, the response to each agent should be evaluated along with the absence of an increase in MAP or the simultaneous up-titration of other agents, and the dose should be adjusted accordingly. Finally, attention should be paid to the titration of adjustable agents to avoid high-dose or prolonged use.^10,11,12^

## Conclusion

VS is a severe and life-threatening condition that continues to challenge anaesthesiologists. When VS is encountered in the perioperative period, the anaesthesiologist should deal with difficulties correctly and effectively. VS is a significant complication that increases morbidity and mortality but is reversible if treated correctly and effectively within the first 6 hours. The use of intraoperative TEE should be considered to establish the diagnosis of VS early and quickly and to rule out other causes in the differential diagnosis. Vasopressors should be started if MAP remains low (<60 mmHg) despite adequate volume administration and optimization of cardiac functions. It is recommended to start NE as a first-line agent to restore and maintain systemic perfusion pressure. An anesthesiologist must consider risk factors, diagnose VS, and manage treatment.

## Figures and Tables

**Table 1:**
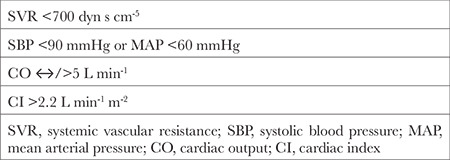
The Diagnostic Criteria of Vasoplegic Syndrome

**Table 2:**
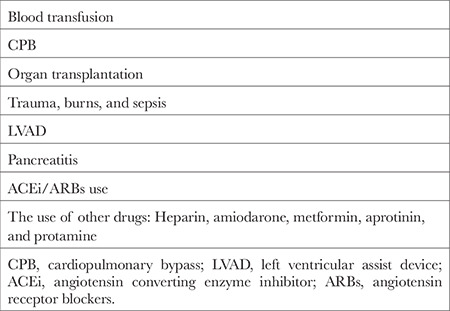
Risk Factors for Vasoplegic Syndrome

**Table 3:**
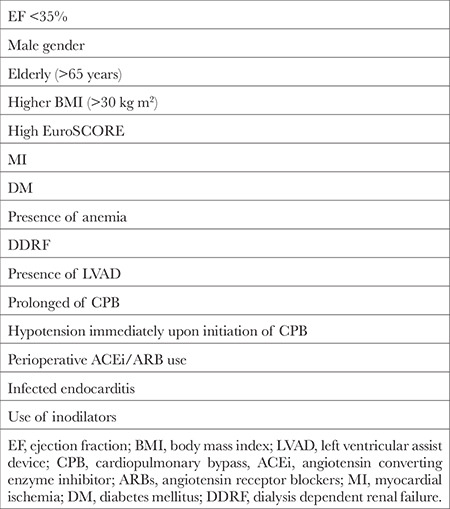
Risk Factors for Vasoplegic Syndrome During Cardiopulmonary Bypass

**Table 4:**
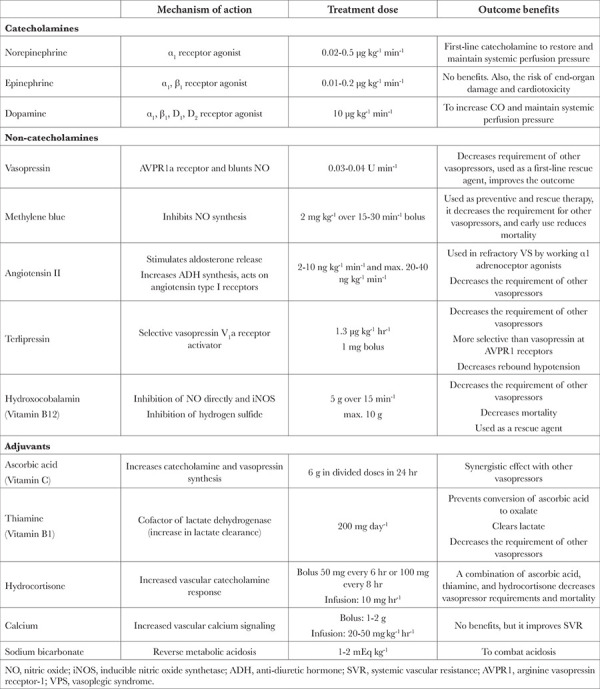
Drugs Used in the Treatment of Vasoplegia Syndrome
